# Associations of Sustainable Development Goals Accelerators With Adolescents’ Well-Being According to Head-of-Household’s Disability Status–A Cross-Sectional Study From Zambia

**DOI:** 10.3389/ijph.2022.1604341

**Published:** 2022-02-25

**Authors:** David Chipanta, Janne Estill, Heidi Stöckl, Lucas Hertzog, Elona Toska, Patrick Chanda, Jason Mwanza, Kelly Kaila, Chisangu Matome, Gelson Tembo, Olivia Keiser, Lucie Cluver

**Affiliations:** ^1^ Institute of Global Health, University of Geneva, Geneva, Switzerland; ^2^ Joint United Nations Programme on HIV/AIDS (UNAIDS), Geneva, Switzerland; ^3^ Institute for Medical Information Processing, Biometry, and Epidemiology, Medical Faculty, Ludwig-Maximilians-University Munich, Munich, Germany; ^4^ Centre for Social Science Research, University of Cape Town, Cape Town, South Africa; ^5^ Department of Social Policy and Intervention, University of Oxford, Oxford, United Kingdom; ^6^ Department of Sociology, University of Cape Town, Cape Town, South Africa; ^7^ Social Work and Sociology, University of Zambia, Lusaka, Zambia; ^8^ Disability Inclusion Project Luapula, International Labour Organisation, Lusaka, Zambia; ^9^ Palm Associates Limited, Lusaka, Zambia; ^10^ Economics and Agricultural Sciences, University of Zambia, Lusaka, Zambia; ^11^ Centre for Evidence-Based Intervention, Department of Social Policy and Intervention, University of Oxford, Oxford, United Kingdom; ^12^ Department of Psychiatry and Mental Health, University of Cape Town, Cape Town, South Africa

**Keywords:** sustainable development goals, accelerators, no poverty, social cash transfers, good health, informal cash transfers, inequalities, social protection

## Abstract

**Objectives:** We examined associations between accelerators (interventions impacting ≥2 SDG targets) and SDG-aligned well-being indicators among adolescents 16–24 years old in Zambia.

**Methods:** We surveyed adults from 1,800 randomly sampled households receiving social cash transfers. We examined associations between accelerators (social cash transfers, life-long learning, mobile phone access) and seven well-being indicators among adolescents using multivariate logistic regressions.

**Results:** The sample comprised 1,725 adolescents, 881 (51.1%) girls. Mobile phone access was associated with no poverty (adjusted Odds Ratio [aOR] 2.08, *p* < 0.001), informal cash transfers (aOR 1.82, *p* = 0.004), and seeking mental health support (aOR 1.61, *p* = 0.020). Social cash transfers were associated with no disability-related health restrictions (aOR 2.56, *p* = 0.004) and lesser odds of seeking mental health support (aOR 0.53, *p* = 0.029). Life-long learning was associated with informal cash transfers (aOR 3.49, *p* < 0.001) and lower school enrollment (aOR 0.70, *p* = 0.004). Adolescents with disabled head-of-household reported worse poverty, good health but less suicidal ideation.

**Conclusions:** Social cash transfers, life-long learning, and mobile phone access were positively associated with well-being indicators. Adolescents living with disabled head-of-household benefited less. Governments should implement policies to correct disability-related inequalities.

## Introduction

Adolescents are a crucial population group in attaining the Sustainable Development Goals (SDGs). Adolescence, ranging from 10 to 24 years, is a formative period to intervene on multiple SDGs. This age range encompasses a broad understanding of adolescence and can be used to target health and development investments across a wide range of settings [1]. Adolescents aged 15–24 years comprise 15.5% (1.21 billion) of the global population, reaching 1.29 billion by 2030 [2]. The rapidly developing physical and mental growth in the transition into adulthood during adolescence, has a strong impact on health and well-being in adulthood [1, 3].

In sub-Saharan Africa, where the adolescent population is growing fast, the potential to improve their well-being is more constrained [2]. The region’s adolescents have high rates of mental health conditions, suicide, HIV, and other diseases [4]. A 10-year-old child is six times more likely to die by age 24 in sub-Saharan Africa than in North America or Europe. Globally, suicide is the second leading cause of death among adolescents aged 15–25 years [4]. Planning suicide and suicidal ideation, defined as a preoccupation with thoughts of killing oneself, among adolescents aged 13–17 years in low-income and middle-income countries were the highest in Africa [5].

Not being in employment, education, or training (NEET) also negatively impacts adolescents’ well-being and successful transition into adulthood [6]. In sub-Saharan Africa, one quarter of adolescent girls (25.9%) and one sixth of boys (15.8%) were NEET in 2019 [7]. Of those employed, the majority (94.9%) were in informal employment and living in extreme poverty on less than US$1.90 a day [7]. Mobile phone use, which could improve adolescent achievement in the SDGs, is also limited in the region [8, 9].

Urgent coordinated government action is needed to accelerate the SDGs for the region’s adolescents, particularly in the context of the COVID-19 pandemic [10]. The United Nations Development Programme (UNDP) defines accelerators as interventions or circumstances that positively impact two or more SDG targets [11]. Studies have shown that combining and re-arranging existing interventions have multiple, large, favorable impacts on adolescents’ mental health, the experience of transactional sex, violence, HIV prevention and treatment, and other SDG-aligned outcomes. For example, social protection, cash transfers, education, safe schools, food security, parenting programmes, role of caregivers, and psychosocial support have been shown to be accelerators [12–14].

The world can only achieve the SDGs if the needs of persons with disabilities are met [15]. More than 1 billion people worldwide are living with disabilities. Most of them are left behind in several SDGs, including SDGs 1 and 2 (poverty and hunger), SDG 3 (health and well-being), SDG 4 (education), SDG 5 (gender equality and empowerment of women and girls), SDG 6 (clean water and sanitation), SDG 8 (employment and decent work), SDG 10 (inequality), and SDG 16 (relating to significantly reducing violence) [15, 16]. On the other hand, social protection, cash transfer programmes include people with disabilities [15], often paired with training (life-long learning) to emphasize or explain programme objectives. The programmes also deliver cash and other services via mobile phone to individuals and households [17]. Social cash transfers (SCT), life-long learning (LLL), and mobile phone access (MPA) could potentially be accelerators that can support adolescents in households headed by persons with disabilities. Therefore, we aimed to test whether SCT, LLL, and MPA fulfill the definition of accelerators in this study, and how they interact with the head-of-household’s disability status in improving the SDG-aligned well-being indicators for adolescents.

## Methods

### Study Context

This cross-sectional study used the data collected from August and September 2019 to evaluate the United Nations Partnership on the Rights of People with Disabilities (UNPRPD) project in Luapula province, 760 km north of Zambia’s capital, Lusaka [18]. The project began in January 2019 and ended in December 2021. It aimed to increase access to HIV, sexual and reproductive health and social protection services among girls and women with disabilities receiving the government of Zambia social cash transfers. The International Labour Organisation implemented the project inclusive of people receiving the social cash transfer, regardless of whether they had a disability. The government of Zambia and UN agencies supported it. The evaluation aimed to assess the project’s impacts, on a range of outcomes including education, employment, mental health, and HIV outcomes. The data for the evaluation was collected from the Kawambwa, Mansa, Nchelenge, and Samfya districts of Luapula province.

### Data Sources and Sample

The sample for the evaluation was from among the poorest people in Zambia. It was drawn from adults aged 16 years—the age of consent in Zambia—or older living in households receiving the government of Zambia SCT. The SCT is Zambia’s flagship social protection programme, started in 2010 to address extreme poverty. In 2019, the SCT reached an estimated 70% of extremely poor households with monthly cash payments to help them afford a meal daily for a month. Households are eligible to receive the SCT if government authorities identify them as extremely poor through standard-of-living measures that also satisfy one or more of the following household criteria [19]:

1. Headed by a woman.
2. Headed by a person aged 65 years or older.
3. Having a member with a disability.
4. Having adult members who are unable to work or support themselves.
5. Hosting orphans and vulnerable children (i.e., any child below 18 years who is living with HIV, has lost one or both parents, or lives in a community affected by HIV).

Eligible households received ZMK90 (US$12) per month or ZMK180 (US$24) if they included a person with a disability. Payments were disbursed every 2 months through a local pay point manager, the post office, or the recipient’s bank account [19].

### Sample Size Calculation

For the evaluation, we calculated a minimum sample size of 1,800 households, drawn from 90 community welfare action committees (CWACs), which are political units. Our sample size calculation assumed a statistical significance (α) of 0.05, power of 80%, and Intra-class Correlation Coefficients (ICCs) (*p*) of 0.01–0.08, and intervention effect (ծ) of at least 0.20 on HIV services, mental health, education, and others [20]. We sampled respondents in two stages. In stage one, we sampled CWACs using proportional probability sampling without replacement, which meant that CWACs with more households and typically with more services would be more likely to be selected. In stage two, from each CWAC, we sampled 25 households.

### Procedures

Trained fieldworkers first obtained and recorded oral informed consent from every respondent aged 16 years or older on electronic tablets with thumbprints for oral and signatures for written confirmation of consent. Participation was voluntary and did not affect their SCT benefits. Participants could refuse to answer questions or withdraw from the study at any point. Non-consent and non-responses to questions were also recorded. Field workers administered a questionnaire in Bemba, the area’s local language, on electronic tablets installed with Open Data Kit software. The head-of-household and all household members aged 16 or older were asked questions on socio-demographic characteristics, self-rated poverty, health and well-being, mental health, school enrollment, disability status, proximity to health facilities, health access restrictions related to disabilities, health services used, receipt of SCT offered by the government, non-governmental organizations and individuals, training received, and MPA.

We derived the questions from piloted and validated tools, including the UNICEF Innocenti tools and Demographic and Health Survey. We translated the questions from English into Bemba. We trained the fieldworkers using role plays to ensure their understanding and standardized administration of the questionnaire. We stored and electronically transferred the data to a secure server. For this study, we included and analyzed responses from all respondents aged 16–24 years from the sampled households.

The study protocol was reviewed by the University of Zambia Humanities and Social Sciences Research Ethics Committee (IRB Approval No. 2019-April-001) and the Ethics Committee in the Canton of Geneva (no. 2019-00500).

### Measures and Variables

Based on our review of the literature, we identified three potential accelerators—SCT, LLL, and MPA—and seven SDG-aligned indicator target outcomes in the data representing the following SDGs: 1.2. No poverty, 1.3.1 Informal cash transfers, 3.0. Good health, 3.4.2. No suicidal ideation, 3.4. Seeking mental health support, 4.1. School enrollment and 10.2.1. No health access restrictions related to disability.

We assessed the head-of-household’s disability status with questions from the Washington Group Short Questions (WGSQ) on disabilities [21] as described in Supplementary Table 1 which shows how the variables used in this study were coded.

### Analysis

We conducted analyses in three steps. First, we explored the socio-demographic characteristics, hypothesized accelerators, and SDG-aligned outcomes by head-of-household’s disability status. Second, we tested for associations between each SDG-aligned outcome and hypothesized accelerators simultaneously using Fisher’s exact test. We then reported crude proportions, 95% confidence intervals (CI), and *p*-values. In multivariate regressions, we controlled for age, sex, head-of-household disability status, proximity to health facility, and district. We corrected for multiple hypothesis testing using the Benjamini, Krieger, Yekutieli (BKY) False Discovery Rate (FDR) Sharpened Qs [22]. We interpreted the FDR adjusted *p*-value as a *p*-value of 0.05, resulting in 5% of significant tests being false positives. Third, we predicted adolescents’ probabilities of experiencing each outcome from no accelerators to cumulative accelerator combinations by head-of-household’s disability status. To do this, we used marginal effects models with the Stata margins command keeping other covariates at their mean values. We reported (graphed) the changes in probabilities for each indicator.

As a sensitivity analysis, we calculated adjusted probabilities of experiencing each outcome from multiple-outcome probit models that correlated the error terms of three potential accelerators in each model, using the mvprobit command in the Stata version 14.1 which we used for analysis, set at 50 random draws. Each regression regressed one of the seven SDG-aligned outcomes for adolescents on the three accelerators, controlling for socio-demographic factors. We clustered analyses at the CWAC level.

## Results

Table 1 shows the sample characteristics of 1,725 adolescents. Overall, 881 (51.1%) were girls and 844 (48.9%) were boys. Their median age was 19 years (interquartile range 17–21). Eight percent (145) of the adolescents lived with household heads with disabilities, about half (51.7%,75) of whom reported at least “a lot” of difficulties in remembering, 29.6% (43) in seeing and 9.6% (14) in self-care. Many socio-demographic characteristics and SDG-aligned targets indicators differed significantly between adolescents living with household heads with and without disability; the three hypothesized accelerators did not differ.

**TABLE 1:** Social demographic characteristics, hypothesized accelerators and SDG-aligned targets by disability status of the household head (Impact of social protection programes on HIV outcomes in Zambia 2019).

	Not disabled		Disabled		*p*-value	Total	%
Variables	*n* = 1,580	%	*n* = 145	%		1725	
Socio-demographic characteristics							
Age (years) 16–19	951	60.2	67	46.2		1,018	59.0
20–24	629	39.8	78	53.8	0.001	707	41.0
Sex, Male	787	49.8	57	39.3		844	48.9
Female	793	50.2	88	60.7	0.015	881	51.1
Distance to nearest the health facility (kilometers) 0–6	1,291	81.7	121	83.4		1,412	81.9
7 and over	242	15.3	19	13.1	0.489	261	15.1
Missing	47	3.0	5	3.4		52	3.0
District Kawambwa	502	31.8	55	37.9		557	32.3
Mansa	311	19.7	22	15.2		333	19.3
Nchelenge	378	23.9	31	21.4		409	23.7
Samfya	389	24.6	37	25.5	0.339	426	24.7
Hypothesized accelerators							
SCT No	157	9.9	15	10.3		172	10.0
Yes	1,408	89.1	128	88.3		1,536	89.0
Missing	15	0.9	2	1.4	0.862	17	1.0
MPA No	1,081	68.4	101	69.7		1,182	68.5
Yes	499	31.6	44	30.3	0.759	543	31.5
LLL No	868	54.9	68	46.9		936	54.3
Yes	697	44.1	75	51.7	0.069	772	44.8
Missing	15	0.9	2	1.4		17	1.0
SDG-aligned target indicators							
SDG 1.2. No poverty Very poor	1,103	69.8	115	79.3		1,218	70.6
Moderately poor	462	29.2	28	19.3	0.012	490	28.4
Missing	15	0.9	2	1.4		17	1.0
SDG 1.3.1 Informal cash transfers No	1,260	79.7	118	81.4		1,378	79.9
Yes	305	19.3	25	17.2	0.561	330	19.1
Missing	15	0.9	2	1.4		17	1.0
SDG 3. Good health Physically sick	1,062	67.2	107	73.8		1,169	67.8
Not sick	503	31.8	36	24.8	0.086	539	31.2
Missing	15	0.9	2	1.4		17	1.0
SDG 3.4. No suicidal ideation Yes	156	9.9	29	20.0		185	10.7
No	1,404	88.9	114	78.6	0.000	1,518	88.0
Missing	20	1.3	2	1.4		22	1.3
SDG 3.4. Seeking mental Health support No	1,087	68.8	100	69.0		1,187	68.8
Yes	479	30.3	43	29.7	0.898	522	30.3
Missing	14	0.9	2	1.4		16	0.9
SDG 4.1. School enrollment No	901	57.0	89	61.4		990	57.4
Yes	678	42.9	56	38.6	0.314	734	42.6
SDG 10. No health access restrictions related to disability Limited	243	15.4	21	14.5		264	15.3
Not limited	1,290	81.6	119	82.1	0.791	1,409	81.7
Missing	47	3.0	5	3.4		52	3.0

*p*-value is for Fisher’s exact test.

Tables 2 and 3 show the associations between each SDG-aligned outcome and hypothesized accelerators. The three hypothesized accelerators—SCT, LLL, and MPA—were significantly associated with no poverty, informal cash transfers, good health, no suicidal ideation, school enrollment, and no health access restrictions related to disabilities when not controlled for socio-demographic covariates. However, only SCT was associated with lower levels of seeking mental health support among adolescents (Table 2). Adolescents with MPA reported higher levels of no poverty (39% versus 23.9%, *p* < 0.001), accessing informal cash transfers (26.6% versus 16%, *p* < 0.001), good health (34.5% versus 30.2%, *p* = 0.042), seeking mental health support (38.4% versus 26.9%; *p* < 0.000), and school enrollment (48.8% versus 39.7%, *p* < 0.001) than those without MPA.

**TABLE 2:** Crude analysis of associations between hypothesized accelerators and SDG-aligned targets (Impact of social protection programs on HIV outcomes in Zambia 2019).

SGD-aligned targets	Hypothesized accelerators, absolute values [proportions], p-value					
	SCT		LLL		MPA	
	Yes	No	Yes	No	Yes	No
1.2. No poverty	442 [28.8%]	46 [26.7%]; 0.318	207 [26.8%]	281 [30.1%]; 0.074	212 [39.0%]	278 [23.9%]; <0.001
1.3.1 Informal cash transfers	323 [21.0%]	7 [4.7%]; <0.001	228 [29.5%]	102 [10.9%]; <0.001	144 [26.6%]	186 [16.0%]; <0.001
3. Good health	489 [31.8%]	50 [29.7%]; 0.258	253 [32.8%]	285 [30.5%]; 0.168	187 [34.5%]	352 [30.2%]; 0.042
3.4. No suicidal ideation	1,381 [90.2%]	137 [79.7%]; <0.001	674 [87.6%]	843 [90.4%]; 0.044	475 [87.6%]	1,043 [89.8%]; 0.102
3.4 Seeking mental health support	447 [29.1%]	75 [43.6%]; <0.001	278 [36.0%]	244 [26.1%]; <0.001	208 [38.4%]	314 [26.9%]; <0.001
4.1. School enrollment	662 [43.1%]	65 [37.8%]; 0.103	302 [39.1%]	424 [45.4%]; 0.005	265 [48.8%]	469 [39.7%]; <0.001
10. No health restrictions related to disability	1,293 [85.8%]	116 [69.9%]; <0.001	613 [82.0%]	795 [86.0%]; 0.014	453 [83.9%]	956 [84.4%]; 0.425

Fisher’s exact test.

**TABLE 3:** Associations between hypothesized accelerators and SDG-aligned targets indicators adjusted for social demographic characteristics (Impact of social protection programs on HIV outcomes in Zambia 2019).

SGD-aligned targets	Hypothesized accelerators (adjusted odds ratios, 95% confidence intervals, *p*-value)		
	SCT	LLL	MPA
1.2. No poverty	1.15 [0.66–1.98], 0.624	0.86 [0.59–1.23], 0.398	2.08 [1.39–3.09], 0.001*
1.3.1 Informal transfers	7.68 [2.56–23.01], 0.000*	3.49 [2.24–5.45], 0.001*	1.82 [1.21–2.74], 0.004*
3. Good health	1.06 [0.55–2.04], 0.859	1.14 [0.85–1.54], 0.379	1.27 [0.89–1.80], 0.184
3.4. No suicidal ideation	1.93 [0.93–3.99], 0.077	0.95 [0.59–1.49], 0.809	0.86 [0.49–1.51], 0.594
3.4 Seeking mental support	0.53 [0.29–0.94], 0.029*	1.34 [0.99–1.80], 0.054	1.61 [1.08–2.40], 0.020*
4.1. School enrollment	1.22 [0.87–1.72], 0.246	0.70 [0.55–0.89], 0.004*	1.65 [1.25–2.18], 0.001*
10. No disability health access restrictions	2.56 [1.35–4.88], 0.004*	0.67 [0.42–1.07], 0.097	0.92 [0.58–1.45], 0.713

Type of test conducted Wald Test. *Statistically significant (*p* < 0.05) after multiple hypothesis testing correction with the FDR, sharpened Qs. adjusted for age, gender, household head disability status, distance to the nearest health facility and district.

After adjusting for age, gender, head-of-household’s disability status, distance from the nearest health facility, and district, all hypothesized accelerators remained associated with two or more SDG-aligned outcomes (Table 3). Good health and no suicidal ideation were no longer associated with any hypothesized accelerator. Having access to a mobile phone was associated with higher odds of no poverty, accessing informal cash transfers, seeking mental support, and school enrollment. SCT were associated with higher odds of informal cash transfers, no health access restrictions related to disability but lower odds of seeking mental health support. LLL was associated with increased odds of accessing informal cash transfers but lower odds of school enrollment.

**FIGURE 1:**
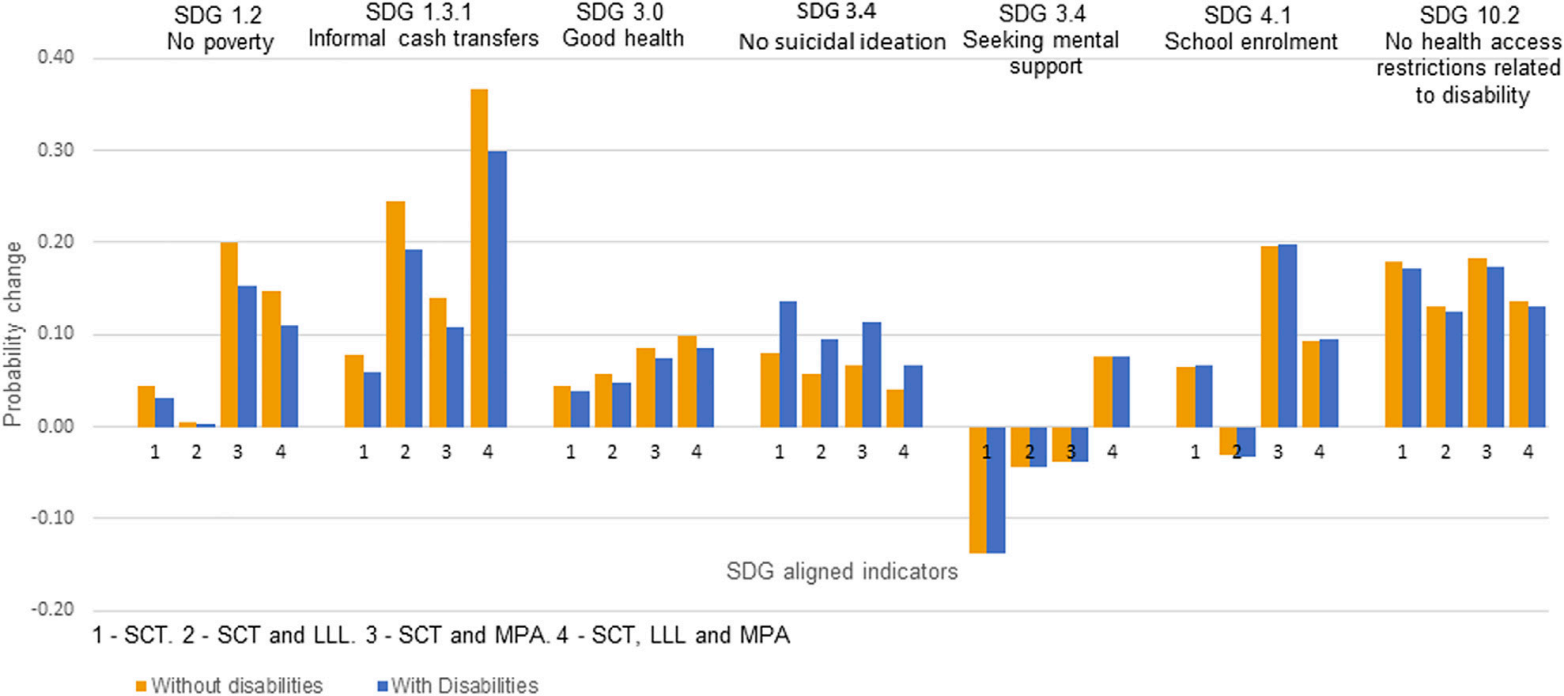
Levels of probability change in SDG-aligned targets indicators outcomes from 1) SCT alone, 2) SCT plus LLL 3) SCT plus MPA, and 4) SCT plus LLL and MPA, stratified by household heads’ disability status—without (blue bars) and with disabilities (Orange bars) (Impact of social protection programs on HIV outcomes in Zambia 2019).

Figure 1 shows the changes in probabilities of experiencing each of the seven SDG-aligned outcomes from potential accelerators compared to no accelerators: 1) SCT alone, 2) SCT plus LLL, 3) SCT plus MPA, and 4) SCT plus LLL and MPA. Results are stratified by disability status of the household head. Potential accelerators were associated with an absolute increase of at least 0.02 in the probability of SDG-aligned target indicators. However, the probability of seeking mental health support was decreased by SCT alone, SCT plus LLL, and SCT plus MPA. The probability of school enrollment was also decreased by SCT plus LLL.

Adolescents with head-of-household with disabilities had lower probabilities of reporting no poverty, accessing informal cash transfers, good health, and no suicidal ideation from no potential accelerators than their counterparts without a head-of-household with disabilities. They further reported lower probability changes from potential accelerators in no poverty, accessing informal cash transfers, good health, and no health access restrictions related to disability. The probability increase in no suicidal ideation from potential accelerators was higher among adolescents living with head-of-household with disabilities. Changes in seeking mental health support and school enrollment did not differ by the disability status of the head-of-household. The greatest probability changes from receiving no potential accelerators to receiving potential accelerators were in accessing informal cash transfers (Figure 1).

**FIGURE 2:**
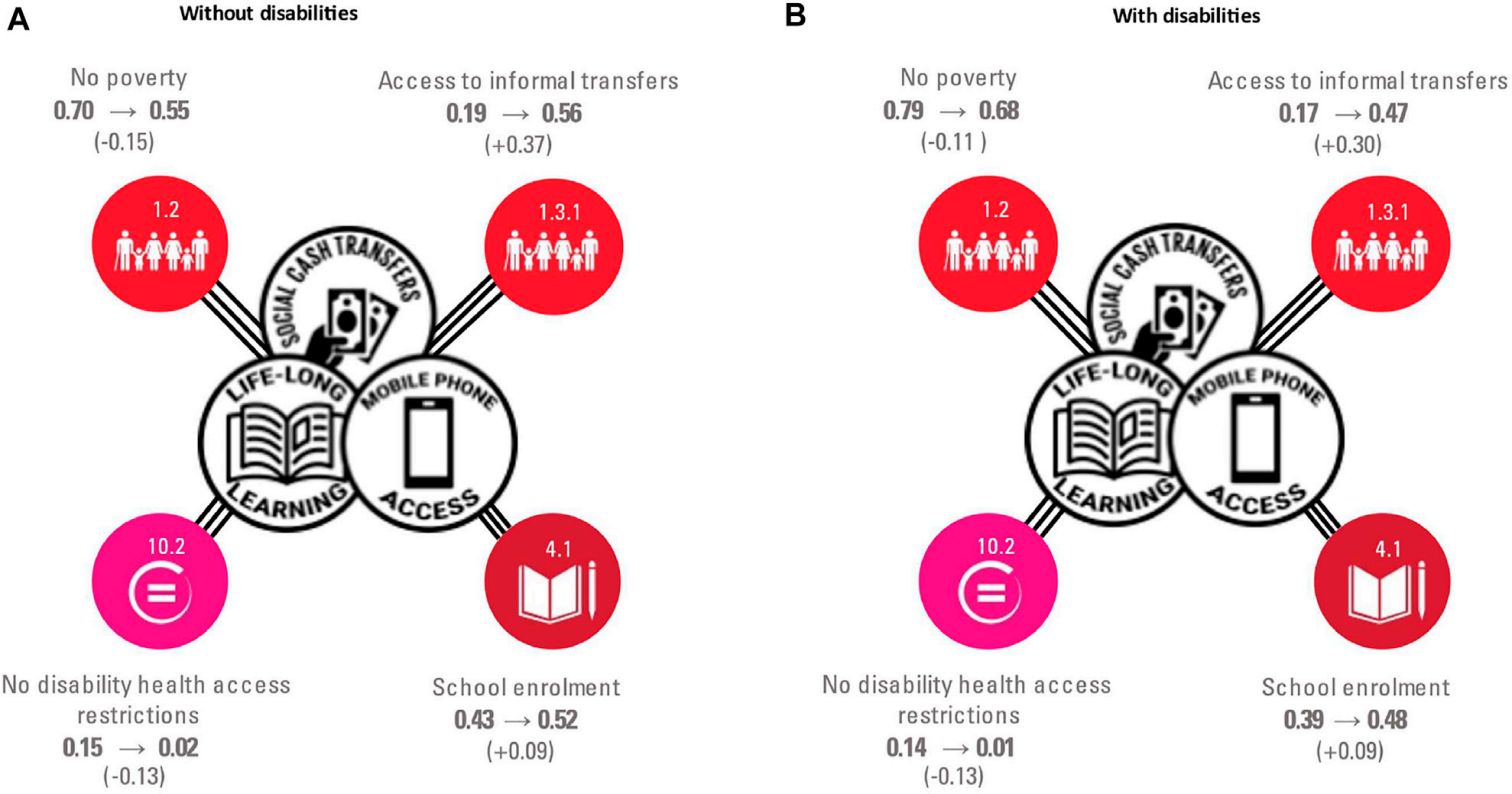
Changes in probability levels of SDG-aligned outcomes for adolescents living with household heads without **(A)** and with disabilities **(B)** from synergies of interventions (Impact of social protection programes on HIV services in Zambia 2019).

Synergies—combinations—of potential accelerators were associated with changes in the probabilities of experiencing levels of SDG-aligned targets indicators outcomes for adolescents living with head-of-household with and without disabilities. A combination of all potential accelerators—SCT, LLL, and MPA—was associated with a 0.15 and 0.11 probability increase in levels of no poverty for adolescents living with head-of-household with and without disabilities; 0.37 and 0.30 probability increase in levels of accessing informal cash transfers, and 0.14 and 0.13 of experiencing no health access restrictions related to a disability, respectively (Figure 2).

The sensitivity analysis results between the models we used and the models that account for correlations between the error terms of the potential accelerators were equivalent. However, the *p*-values were lower in the outcome models accounting for the correlation between potential accelerators (Supplementary Table 2).

## Discussion

This study examined associations between potential accelerators—SCT, LLL, and MPA—and SDG-aligned well-being indicator targets—1. no poverty; 1.3.1. SCT, informal cash transfers; 3.4.2. good health; 3.4. no suicidal ideation, seeking mental health support; 4.1 school enrollment; and 10.2.1 no health access restrictions related to disability—among adolescents in Zambia. We found high potential for improving vulnerable adolescents’ SDG-aligned well-being by combining SCT, LLL, and MPA interventions. Our findings fit within an emerging body of evidence confirming that SCT, LLL, and MPA are accelerators for adolescents [8, 10, 13]. This study further found that adolescents benefited unequally depending on the disability status of their head-of-household and that combining existing interventions may not overcome these inequalities.

Several studies, conducted in Zambia and elsewhere, confirm our findings that SCT were associated with multiple SDG-aligned target outcomes such as higher levels of informal cash transfers, no health access restrictions related to disability, and lower levels of seeking mental health support. Studies conducted in Zambia show that SCTs reduced relative poverty, increased schooling among adolescents and women’s satisfaction regarding their children’s well-being [23, 24]. SCT also increased material well-being (children’s material needs met), food security, and asset ownership [25]. In sub-Sahara Africa and elsewhere, SCT have been shown to increase psychological well-being, and decrease relative and absolute poverty [26, 27]. In our study, receiving SCT alone was associated with a substantial decrease in seeking mental health support. Combining SCT with LLL or MPA was associated with greater reductions in seeking mental health support. This result suggests that households’ lack of money, LLL opportunities, and MPA may have necessitated respondents to seek mental health support. Providing SCT, LLL, and MPA interventions may be vital for addressing the mental support needs of adolescents living in poverty.

In our study, however, SCT were not associated with good health; neither were MPA or LLL. This result fits within a body of evidence showing that cash transfers have positive, complex, and mixed effects on health. A review of 56 studies from low- and middle-income countries found that cash transfers increased dietary diversity, access, and utilization of health services but had little impact on children’s anthropometric measures [26]. In high-income countries, self-rated health, chronic health conditions, and mortality for cash transfer recipients were worse than among non-recipients. On the contrary, in the United States, cash transfers were associated with improved self-rated health [28]. One reason why SCT, MPA, and LLL in our study were not associated with good health could be that physical illness was widespread among our sample. Two-thirds (67.8%, *n* = 1,169) of adolescents reported physical illnesses. Another is that malaria is endemic in the study area [29]. SCT, MPA, and LLL alone might have been insufficient to resolve these illnesses. Innovative prevention and treatment of malaria and other illnesses, combined with SCT, MPA, and LLL could have a positive cumulative impact. They should be implemented.

Contrary to views that cash transfers and other public transfers reduce informal transfers [30], our study found the opposite result; SCT were associated with increased receipt of informal cash transfers [31, 32]. One explanation for our study’s finding is that the process of receiving SCT may have identified households in need of financial and material support, linked them to that support, strengthened mutual trust, and increased social inclusion and solidarity among them [31–33]. Another explanation is that our study did not include pensions and social security transfers, which were analyzed in a study with contradictory findings [30]. Pensions and social security transfers derived from mandatory savings that employees make tend to be larger than SCT. In addition, pensions and social security transfer recipients may be wealthier, making them less likely to be perceived as in need of informal cash transfers [31].

The positive associations found in our study between MPA and no poverty, informal cash transfers, and school enrollment are also supported by evidence [8, 34]. Access to mobile phones can promote adolescents’ well-being and expand their social networks and personal growth opportunities [8]. Social protection and cash transfers are being delivered to adults via mobile phones, alongside electronic vouchers, bank accounts, and other payment systems [35]. Mobile phone use also enables access to vital services [8, 34]. Informal financial transfers make up the bulk of mobile phone transfers in sub-Sahara Africa [36]. The negative association found in our study between MPA and seeking mental health support suggests that lack of mobile phone access may be mentally distressing for adolescents. One reason is that they may miss out on informal cash transfers and other services to improve their well-being [8, 34, 36]. Providing mobile phones to households which do not have them is being implemented and can help improve adolescents’ access to social protection, cash transfers, and mental health support [35].

In our study, LLL’s associations—increase in informal cash transfers, and reductions in odds of seeking mental health support and school enrollment—are more limited than those of SCT and MPA, but no less critical to improving adolescents’ well-being. LLL reinforces and complements the objectives of social protection and cash transfer programs. LLL often bring participants together, potentially increasing their social networks—mitigating the need for mental health support—and increasing opportunities for informal cash transfers. LLL is unlikely to have attracted adolescents away from school. Two-thirds of adolescents in our sample were already not attending school. LLL and the skills it provides can be beneficial to them [37].

Adolescents in our sample did not evenly benefit from SCT alone, from SCT with LLL and MPA, or LLL and MPA although they benefited from these potential accelerators. Adolescents living with head-of-household with disabilities reported lower benefits from these potential accelerators in the areas of no poverty, informal cash transfers, good health, and no disability health access restrictions than their peers living with head-of-household without disabilities. Previous studies, including a systematic review, support this finding, showing that living with a household member with a disability entails high costs and poverty implications for the household [38, 39]. These households must spend as much as 26% more to obtain an equivalent standard of living compared to those without disabilities [38, 39]. Adolescents in our sample living with a head-of-household with disabilities reported themselves poorer, with much more diminished resources, and saddled with caregiving responsibilities that adversely affect their well-being compared to their peers in household headed by a person without disabilities. However, adolescents living with head-of-household with disabilities reported greater benefits from accelerators in no suicidal ideation compared to their peers with head-of-household without disabilities. This result suggests that a head-of-household’s disability status may mitigate suicidal ideation among adolescents. Such adolescents may have benefited from parental supervision during caregiving, which is known to be protective against suicidal behavior [40]. However, this study did not investigate the role of the head-of-household’s disability status on adolescents’ suicidal ideation.

Overall, accelerators appear to positively impact adolescents’ well-being. However, adolescents living with head-of-household with disabilities were doing worse than their peers both before and after the accelerators. New interventions focused on households living with a head-of-household with a disability may be required. These could include attention to adolescent–parent relationships, and increased SCT, MPA, LLL, psychosocial, mental health, and financial support to offset disability-related inequalities [38].

### Limitations

In this study, we could not attribute causation due to the study’s cross-sectional nature. However, studies show that disability is at the root of multiple deprivations [15, 38]. It is important to note that we could not generalize the results outside the study area and population group. However, many countries in sub-Saharan Africa are implementing similar programmes and could find our results useful in their contexts. We performed a complete sample analysis due to the low prevalence of disability in our sample, which might have missed nuanced differences experienced by adolescent girls compared to boys. We did not have variables on occupation of the head-of-household, and others that could affect household dynamics, including adolescents’ well-being. We did not calculate the sample size for the associations explored in this study but for the parent study to evaluate the impact of the UNPRPD project. Neither did we impute the missing data because it was less than 5%.

### Conclusion

Our study found multiple and substantial favorable associations between accelerators—SCT, LLL, and MPA—delivered individually and in combination, with SDG-aligned well-being among adolescents living in poverty. However, adolescents living with a head-of-household with disabilities benefited less. Policies should be adapted to include new interventions to correct disability-related inequalities. More research is needed to understand interventions to address disability-related inequalities affecting adolescents living with disabled head-of-household.
