# Supplementary material for: Associations of Sustainable Development Goals Accelerators With Adolescents’ Well-Being According to Head-of-Household’s Disability Status–A Cross-Sectional Study From Zambia
Source: Int J Public Health. 2022 Feb 25;67:1604341. doi: 10.3389/ijph.2022.1604341 (PMC8916123; doi:10.3389/ijph.2022.1604341)
Supplement: Supplementary file 1 [file Table1.DOCX]

| **Supplementary Table 1: Variable description and their measurement (Impact of social protection programmes on HIV outcomes in Zambia 2019)** | | |
| --- | --- | --- |
| **Variables** | **Description** | Measurement |
| **Socio-demographic characteristics** | |  |
| Age | Age in linear years was categorized 16 to 19, 20 to 24 years. | Categorical |
| Sex | Biological sex defined from the question are you male, or female? Coded male, female. | Binary |
| Distance to nearest the health facility (kilometres) | Proximity to health facility was defined from the question how far the nearest health facility in kilometres is, categorized less than 7 km or more than 7 km. | Categorical |
| District | Was a dummy variable that captured the name of the disctrict, Kawambwa, Nchelenge, Mansa, or Samfya. | Categorical |
| **Hypothesized accelerators** | |  |
| Social Cash Transfers (SCT) | SCT provided by the government was defined with the question: “During the past 12 months, has the respondent or any household member received money or goods, including food, clothing, livestock, or medicines from any of the following government programmes, social cash transfers and other government transfers?” (Other government transfers combined respondents or their household’s receipt of school uniforms, scholarships, food security pack, school feeding, and farm input subsidy), coded no, yes. | Binary |
| Mobile Phone Access (MPA) | Defined with the question MPA with the question “What phone number is used at this house?” (Response options were no phone, phone number)” coded no for no phone, yes for phone number. | Binary |
| Life Long Learning (LLL) | LLL, combined participation in government offered training on HIV, disability, gender-based violence, human rights, sexual and reproductive rights, job skills, social protection and economic empowerment derived from the question: “During the past 12 months, have you or any of the household members received any training provided by the government on general health, food and nutrition, sexual and reproductive rights, HIV, human rights, and gender-based violence, social protection, job skills, and economic empowerment?” coded yes if the participant responded to have participated in any of the training, otherwise no. | Binary |
| **SDG aligned target indicators** | |  |
| SDG 1.2. No poverty Very poor | No poverty was defined with the question: “Do you consider your household to be nonpoor, moderately poor, or very poor?” coded very poor, moderately poor. | Binary |
| SDG 1.3.1 Informal cash transfers | Informal cash transfers were defined with the question: “During the past 12 months, has the respondent or any household members received money or goods, including food, clothing, livestock, or medicines from individuals who are not part of the family or non-governmental organizations?” coded no, yes. | Binary |
| SDG 3. Good health | Good health was defined with the question: “Have you been sick or injured in the last two weeks?” coded physically sick, not sick. | Binary |
| SDG 3.4. No suicidal ideation | No suicidal ideation was defined with the question: “Did you have thoughts of hurting or killing yourself? coded yes, no. | Binary |
| SDG 3.4. Seeking mental support | Seeking mental support with the question “What health facility or other institutions or persons did you see for any of the identified mental health issues?” coded no did not see; yes saw. Seeking mental support proxied having mental health problems and seeking help to resolve them. | Binary |
| SDG 4.1. School enrolment | School enrolment combined the responses from the question: “Are you currently attending school? (Check relevant choice) nursery/pre-school, other grades full-time, other grades part-time, community school, full-time, correspondence, adult literacy class, tertiary school” coded no, yes. The proportion of adolescents currently in school versus those not in school for 20 to 24-year-olds, coded no, yes. | Binary |
| SDG 10. No health access restrictions related to disability | No health access restrictions related to a disability with the question: “Are you limited in accessing health services because of your impairment?” coded limited, not limited. | Binary |
| Missing | refused to respond was coded as missing | Missing |
