# Supplementary material for: Associations of Sustainable Development Goals Accelerators With Adolescents’ Well-Being According to Head-of-Household’s Disability Status–A Cross-Sectional Study From Zambia
Source: Int J Public Health. 2022 Feb 25;67:1604341. doi: 10.3389/ijph.2022.1604341 (PMC8916123; doi:10.3389/ijph.2022.1604341)
Supplement: Supplementary file 2 [file Table2.DOCX]

| Supplementary Table 2: Associations between hypothesized accelerators and SDG-aligned targets using probit models (Impact of social protection programs on HIV outcomes in Zambia 2019) | | | |
| --- | --- | --- | --- |
| SGD-aligned targets | Hypothesized accelerators, Coefficient [95% CI], P-value | | |
|  | SCT | LLL | MPA |
| 1.2. No poverty | 0.15 [-0.08 - 0.37], 0.197 | -0.14 [-0.27 - -0.01], 0.043 | 0.44 [0.30 - 0.57], <0.001 |
| 1.3.1 Informal cash transfers | 0.80 [0.44 - 1.17], <0.001 | 0.66 [0.51 - 0.81], <0.001 | 0.35 [0.20 - 0.50], <0.001 |
| 3. Good health | 0.14 [-0.08 - 0.36], 0.215 | 0.04 [0-.09 - 0.17],0.549 | 0.13 [-0.01 - 0.26], 0.064 |
| 3.4. No suicidal ideation | 0.44 [0.20 - 0.69], <0.001 | -0.17 [-0.33 - -0.01], 0.048 | -0.08 [-0.26 - 0.09], 0.342 |
| 3.4 Seeking mental health support | -0.44 [-0.65 - -0.23], <0.001 | 0.29 [0.16 - 0.42], <0.001 | 0.31 [0.18 - 0.45], <0.001 |
| 4.1. School enrollment | ’0.17 [-0.04 - 0.40], 0.117 | -0.23 [-0.36 - -0.1], <0.001 | 0.31 [0.17 - 0.45], <0.001 |
| 10. No health restrictions related to disability | 0.65 [0.42 - 0.88], <0.001 | -0.22 [-0.37 - -0.08], 0.003 | -0.02 [-.18 - 0.13], 0.776 |
| Probit models accounting for correlation between error terms. Adjusted for age, gender, household head disability status, distance to the nearest health facility and district | | | |
